# Prevalence of Depressive Symptoms and Its Correlates among Male Medical Students at the University of Bisha, Saudi Arabia

**DOI:** 10.3390/healthcare12060640

**Published:** 2024-03-12

**Authors:** Abdullah M. Alshahrani, Mohammad S. Al-Shahrani, Elhadi Miskeen, Muffarah Hamid Alharthi, Mohannad Mohammad S. Alamri, Mohammed A. Alqahtani, Mutasim E. Ibrahim

**Affiliations:** 1 Department of Family and Community Medicine, College of Medicine, University of Bisha, P.O. Box 731, Bisha 61922, Saudi Arabia; mualharthi@ub.edu.sa (M.H.A.);; 2 Department of Family Medicine, Armed Forces Hospital South Region, Khamis Mushait 62413, Saudi Arabia; malshahrani124@hotmail.com; 3 Department of Obstetrics and Gynecology, College of Medicine, University of Bisha, Bisha 61922, Saudi Arabia; emiskeen@ub.edu.sa; 4 Healthcare Delivery Department, Third Health Cluster, Ministry of Health, Riyadh 12382, Saudi Arabia; moaalqahtani@moh.gov.sa; 5 Department of Basic Medical Sciences, College of Medicine, University of Bisha, Bisha 61922, Saudi Arabia; meibrahim7@gmail.com; 6 Department of Medical Education, College of Medicine, University of Bisha, Bisha 61922, Saudi Arabia

**Keywords:** depression, correlates, medical students, Saudi Arabia

## Abstract

Background: Identifying the potential factors of depression among medical students is the first step towards academic excellence and future safe medical practice. Methods: A cross-sectional study was conducted from December 2019 to February 2020 at the University of Bisha, College of Medicine (UBCOM), Bisha Province, Saudi Arabia. Male medical students from year one to year six were involved. A self-administered questionnaire was used to collect data about students’ socio-demographic and academic characteristics. The Arabic version of the PHQ-9 scale with a score of ≥10 was used to identify depression. Logistic regression analysis was used to assess the prevalence and correlates of depression. Results: Of the 190 male students enrolled, 26.8% had depressive symptoms, of whom 45.1% were experiencing moderate to severe symptoms. The significantly highest depression rate was found among the second-year students, at 43.8% (OR = 2.544; 95% CI 1.178–5.714; *p* = 0.018), and the lowest rate was found among year one students, at 8.9% (OR = 0.203; 95% CI 0.075–0.560; *p* = 0.002). Univariate regression revealed a significant correlation between depression and dissatisfaction with family income, loss of family members, having psychological illness, difficulties in personal relationships, regretting studying medicine, failure in an academic year, a lower grade than expected, conflict with tutors, lack of college facilities and heavy academic load. In multivariate analysis, loss of family members (AOR = 3.69; 95% CI 1.86–7.413), difficulties in personal relationships (AOR = 2.371; 95% CI 1.009–5.575), regretting studying medicine (AOR = 3.764; 95% CI 1.657–8.550), and failing an academic year (AOR = 2.559; 95% CI 1.112–5.887) were independently correlated with depression. Conclusions: The study concluded that medical students at UBCOM experience depressive symptoms associated with various risk indicators. Optimizing the educational and social environment and infrastructure facilities at UBCOM might promote students’ mental health and well-being.

## 1. Introduction

Depression is a common mental disorder expressed by loss of interest and pleasure, persistent sadness, decreased energy, inability to carry out daily activities and low concentration [1,2]. This disorder can affect all health aspects of the individual, including physical condition, mental and academic performance and social life [3]. Globally, about 350 million individuals suffer from depression and almost 3.2% of them express having an episode of depression at least once in their lives [4]. Medical students experience a higher degree of psychological morbidity ranging from stress, interpersonal problems and suicidal ideation to psychiatric disorders, compared with other populations [1]. However, in the era of the Coronavirus disease 2019 (COVID-19) outbreak, medical students were found to be highly vulnerable to mental health problems. This vulnerability could be attributed to the increased risk of infection, significant changes in lifestyle, strict confinement, and disruptions in education caused by the pandemic [5].

The potential effects of depression on medical students include impairment of functioning in classroom and patient care settings that would negatively reflect community health [1,6]. Worldwide studies have reported high rates of depressive disorders among medical students, which affect their health and academic achievements [4,7]. A systematic review determined that the pooled prevalence of depression was 24.2% among male medical students around the globe [8]. A recent investigation indicated that depressive symptoms are common among male medical students and interns at Al-Baha University, in the south of Saudi Arabia [9]. Some studies in Saudi Arabia have identified a high frequency of depressive symptoms among medical students [2,10,11]. Despite there being more than 30 medical schools across Saudi Arabia [12], there are no available data estimating the rates of depression among students in many medical schools. Continuous screening of depressive symptoms among medical students could help to take further appropriate interventional measures to prevent the complications of depression among these future medical professionals [8].

It is widely accepted that medical students are at risk of depression due to frequent academic demands, poor learning environments, and an inability to cope with stressful situations in clinical practice [1,9]. Also, many social life indicators, physical health, a history of psychological illness, and financial concerns make medical students more susceptible to depression [2,13]. Studies have identified several factors associated with higher odds of depression among medical students which include age, gender, physical health, social support, conflicts with teachers, personal events, finances, education, sleep, diet, socioeconomic status, family history, and emotional abuse [9,14,15,16,17]. In a descriptive cross-sectional study conducted among medical students in Canada, a high prevalence of major depressive disorder was reported among those who lacked sufficient social support from friends and family [15]. A multicenter cross-sectional study conducted in 12 Italian medical schools found factors associated with an increased risk of depressive symptoms, including being female, older age, poor economic status, lack of exercise, having relatives with psychiatric disorders, a personal chronic disease, negative perceptions of medical school, unsatisfying friendships with classmates and being worried about not measuring up to the profession [18]. Similarly, a cross-sectional study carried out among 1103 medical students at a middle-sized German university found that spending time with partners, friends, family, hobbies and exercise and confiding in classmates about worries were main factors associated with less depressive symptoms. However, the significant predictors for depressive symptoms were neuroticism, above all, insufficient emotional support, eating irregular meals, use of medication or drugs to calm down, and mental overload [19].

Medical students who experience early onset of depression face an elevated risk of issues such as substance abuse and suicidal behavior [20]. Therefore, medical students at extreme risk of depression need serious attention to identify and tackle the possible factors that could impair their academic path and future professional career [1]. Substantially identifying depressive symptoms and their associated factors among medical students is the first step towards academic excellence and safe medical practice [21].

The research on depressive symptoms among medical students has indeed been explored by various international researchers, reflecting the acknowledgment of the mental health challenges faced by this specific demographic. Numerous studies have consistently reported high rates of depression among medical students globally [16,22], emphasizing the need for a deeper understanding of the contributing factors and tailored interventions. The focus on male medical students at the University of Bisha sets this research apart. Although many studies explore depressive symptoms in medical students, this research narrows its scope to a specific gender and institution. This specificity allows for a more targeted analysis of factors that may be particularly relevant to male medical students at this university. The present study aimed to determine the prevalence of depression and its associated factors among male medical students at the University of Bisha, Bisha province, in southwest Saudi Arabia.

## 2. Materials and Methods

### 2.1. Study Design and Setting

A cross-sectional study was conducted during a period from December 2019 to February 2020 at the University of Bisha, College of Medicine (UBCOM) in Bisha province, in southwest Saudi Arabia. Male medical students (n = 201) at different academic levels from year one to year six were enrolled in the study.

UBCOM is a new medical school in Saudi Arabia, which was established in 2014 to contribute to improving the health care status in the country. The educational program in UBCOM is an integrated curriculum that utilizes problem-based learning strategies blended with various student-centered activities [23,24]. The medical curriculum in UBCOM is implemented from year two to six, whereas the first year is a preparatory phase [25,26].

### 2.2. Procedures of Sampling Collection

The data were gathered through a self-administered questionnaire comprising two distinct forms.

The first part of the questionnaire gathered information about participants’ sociodemographic characteristics (such as age, residency, parents’ education levels, and family income), sociopsychological status (including a history of psychological illness, social activity, and personal relationships), and academic information (such as academic performance, load, failure, and grade average). These factors were selected based on an extensive review of the literature, which has shown that they contribute to depressive symptoms among medical students [4,9,14,27,28,29]. However, the first section of the questionnaire was coded to test hypotheses from the literature and served as independent variables.

The second part of the questionnaire includes the Patient Health Questionaire-9 (PHQ-9) to assess depression among the participants.

### 2.3. Sampling Technique

A survey was conducted on medical students using a simple random sampling approach. The researchers distributed the questionnaire form to the students who had volunteered for the study during class. Cover letters described the purpose of the survey distributed with questionnaires in the presence of the researchers to clarify any queries or doubts. Participating in the study was voluntary, with no influence on their educational progress. However, students who attended the session were free to decline participation in the survey at any stage.

All students, including those with mental health problems and chronic illnesses such as diabetes, hypertension or asthma were included in the study. Students who were absent during the survey or not registered for the current academic year were excluded from the study.

### 2.4. Description of PHQ-9 Instrument

A translated Arabic version of the PHQ-9 scale was used for the study. This version has been previously tested for its validity and reliability as a suitable tool for detecting depressive symptoms in a Saudi Arabian context. The Arabic version of the PHQ-9 showed good internal consistency with Cronbach’s alpha of 0.857. Inter-item correlations range between 0.177 and 0.648 [30]. The PHQ-9 is a self-administered instrument consisting of nine items (from 1 to 9), each based on a four-point Likert-type scale that scores for the presence of depressive symptoms from zero to three as follows: “not at all”, “several days”, “more than half the days”, and “nearly every day”, respectively. Participants were diagnosed with depression if their responses to the following depressive symptoms criteria were met and existed for two weeks. Therefore, major depression was accounted for if the answer to items number 1 or 2 and 4 or more of the remaining PHQ-9 items recorded at least “more than half the days”. Based on these criteria, a PHQ-9 score of ≥10 was used as a diagnostic cutoff point for depressive symptoms, as previously recommended in the literature [30,31].

### 2.5. Statistical Analysis

The information was input and examined utilizing the Statistical Package for the Social Sciences (version 24) (Armonk, NY, USA: IBM Corp.). A descriptive analysis was used to summarize data in terms of distributions, frequencies and proportions. A chi-square test was used to compare the proportion of depressive symptoms between students at different academic levels. Univariate logistic regression was performed to identify the correlates of depressive symptoms and presented as crude odds ratios (COR) with 95% confidence intervals (CI). Then, all independent variables with *p* < 0.05 values were retained for multivariate analysis. Multivariate logistic regression was employed to ascertain independent associations with symptoms of depression and presented as adjusted odds ratios (AOR) with 95% CI. The AOR with 95% CI and a *p*-value of 0.05 was used to determine the final model. Prism version 7 (GraphPad Software, La Jolla, CA, USA) was used for plot graphs.

## 3. Results

### 3.1. Sociodemographic Characteristics of Participants

Out of the 201 medical students enrolled in UBCOM, 190 (94.5%) responded to the survey, 7 declined to participate and 4 provided incomplete information. Of the 190 participants, 45 (23.7%) were from year one, 32 (16.8%) were from year two, 41 (21.6%) were from year three, 25 (13.2%) were from year four, 30 (15.8%) were from year five and 17 (8.9%) were from year six. The age of participants ranged from 18 to 25 years, with a mean of 21.1 ± 2.0.

Of the total number of students (n = 190) enrolled, 118 (62.1%) were from Bisha province and the majority (75.3%) were living with their families or friends. Most of the participants were satisfied with their family income (86.3%) and reported a stable parental relationship (88.9%). The minority of students were recorded as having a psychological illness or having a family member with a psychological illness (11.6%) and losing family members during the last month (12.1%). Students perceived limited time for social activities, recorded as 77.4%, whereas 23.7% reported difficulties in personal relationships and 24.2% were using a stimulant (tobacco, khat, alcohol and drug addiction). With regards to educational aspects, 25.8% of the students regretted studying medicine, 30.0% failed an academic year, 69.5% suggested having a lower grade than expected, 80.5% reported a lack of college facilities, 65.5% complained about the heavy academic load and 14.2% had a conflict with tutors. The detailed demographic characteristics of the participants are shown in Table 1.

### 3.2. Prevalence of Depression

**Figure 1:**
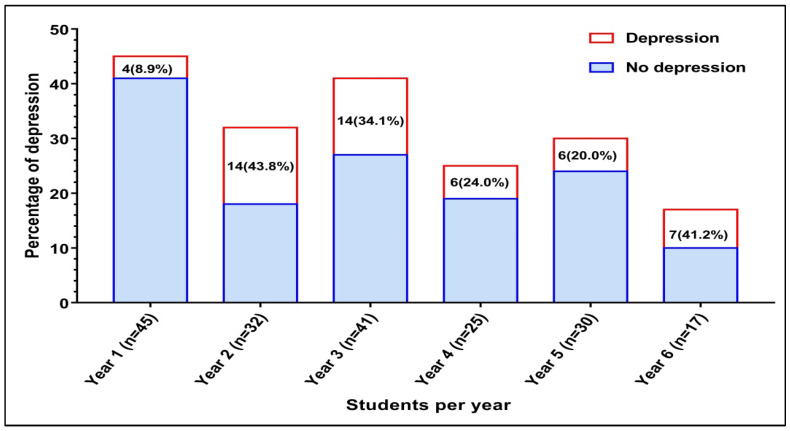
Frequency of depression among medical students by academic year.

**Figure 2:**
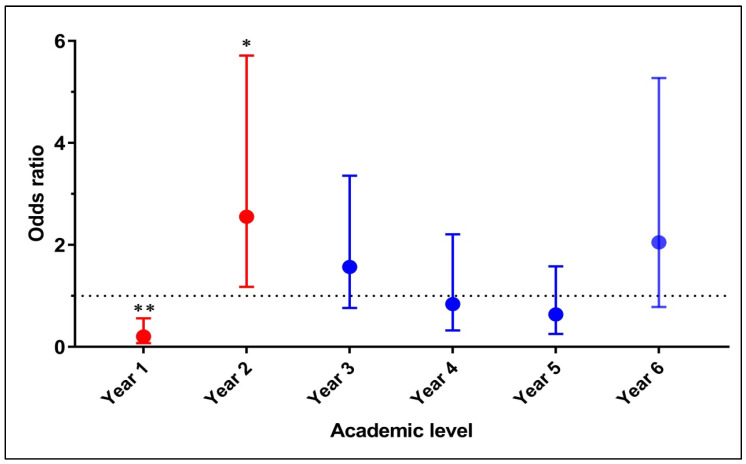
Odds ratio (95% confidence interval) for depressive symptoms among students at each level of study compared to students at other academic levels. ** *p* value < 0.02; * *p* value < 0.05.

The prevalence of depressive symptoms using PHQ-9 (score ≥ 10) was 26.8% (51/190). Of these cases, 54.9% (n = 28) had moderate to severe symptoms, 53.5% (n = 15) had severe symptoms, and 46.4% (n = 13) had moderate symptoms. The prevalence of medical students with depressive symptoms by year of study varied from 8.9% to 43.8% as presented in Figure 1. The highest prevalence rate was found amongst the second-year students, the sixth-year students and the third-year students. However, there were statistically significant differences in depressive symptoms by year of study, as shown in Figure 2. A significant depression rate was found among the second-year students compared to students from all other academic years (OR = 2.544; 95% CI 1.178–5.714; *p* = 0.018). The significantly lowest depression rate was reported among year one students compared to other students (OR = 0.2034; 95% CI 0.075–0.560; *p* = 0.002).

### 3.3. Factors Associated with Depressive Symptoms

Table 1 and Table 2 display the univariate and multivariate analysis of factors associated with depressive symptoms. In univariate analysis, the risk of depressive symptoms was found to be significantly increased among students with the presence of the following factors: dissatisfaction with family income (COR = 2.747; 95% CI 1.173–6.432), loss of their family members (COR = 2.91; 95% CI 1.193–7.101), having a psychological illness, or having a family member with psychological illness (COR = 2.581; 95% CI 1.039–6.413), having difficulties in personal relationships (COR = 2.942; 95% CI 1.447–5.981), regretting studying medicine (COR = 5.245; 95% CI 2.583–10.650), failing an academic year (COR = 2.537; 95% CI 1.293–4.975), receiving a lower grade than expected (COR = 3.020; 95% CI 1.316–6.928), having a conflict with tutors (COR = 2.544; 95% CI 1.098–5.892), perceived lack of college facilities (COR = 2.751; 95% CI 1.008–7.508), and feeling a heavy academic load (COR = 2.310; 95% CI 1.092–4.888) (Table 1). Based on the retention of the significant relationships for the multivariate analysis, the independent correlates of the depressive symptoms were loss of family members during the last month (AOR = 3.69; 95% CI 1.86–7.413) and difficulties in personal relationships (AOR = 2.371; 95% CI 1.009–5.575), regretting studying medicine (AOR = 3.764; 95% CI 1.657–8.550) and failing an academic year (AOR = 2.559; 95% CI 1.112–5.887) (Table 2).

## 4. Discussion

The present study determined the prevalence and correlates of depressive symptoms among medical students at UBCOM in Bisha province in the southern region of Saudi Arabia. The overall prevalence of depression among students was 26.8%. This finding was higher than that (16.2%) reported among male medical students at King Faisal University in the eastern region of Saudi Arabia in 2007 [11]. On the other hand, high prevalence of depression has been reported among male medical students in Albaha University in the neighboring city of Albaha (53.8%) [9]. Moreover, recent studies conducted in Saudi Arabia have reported a high prevalence of depression, with varying rates. For instance, a study conducted among medical students at King Khalid University found a high prevalence rate of depression (88.9%) [17]. Similarly, the prevalence of depression among medical students at Sulaiman AlRajhi Colleges, Al-Qassim was reported to be 42.1% [27]. In other studies conducted elsewhere, similarly high prevalence rates of depression have been reported among medical students, such as 48.4% in Yemen [32] and 43.6% in Namibia [16]. Such figures coupled with our present finding showed an increase in the prevalence of depression among medical students in recent years. Research has shown that medical students worldwide experienced a high prevalence of depression, anxiety, and sleep disorders during the COVID-19 pandemic. In a meta-analysis and systematic review evaluating the global prevalence and risk factors of mental problems, Peng et al. demonstrated a high prevalence and risk factors for mental problems during COVID-19, calling for mental health services [5].

Comparing the level of depression by the academic year, the second-year students scored the highest depression rate. This result is consistent with previous studies using different assessment tools for depression [33]. For instance, high rates of depression have been determined by the PHQ-9 scale among second-year medical students in Korea [34] and Malaysia [35]. These observations could be explained by the fact that medical students could face a more complex curriculum in the second year. In UBCOM, the massive content of the medical curriculum is presented in year two, where the students learn comprehensive knowledge of the human body’s structure, function and biochemical basis in health and disease [25]. Likewise, increasing the level of depression among students during the early stages of medical school has been reported in several studies. Vankar et al. determined that the prevalence of self-identified depression was significantly higher in the first year and second years compared to the third and fourth years [33]. In a longitudinal study, Roh et al. have suggested that depression rates increase during the first year and then reach the peak level during the second year, followed by a gradual decline during the later years of medical school [36]. A recent study in Saudi Arabia conducted among medical science students found that depression starts to escalate from the pre-professional year, reaches a peak in the third professional year, and then decreases in the final year of graduation [2].

Interestingly, our sixth-year students recorded the second-highest rate of depression. This might be due to the increasing demand for clinical training and new concerns and responsibilities of students during such academic levels. A recent study highlighted several stressful situations like using psychometric skills, applying clinical knowledge in real-life situations, trauma exposure, understanding the role, and regulating clinical settings during their clerkship [37]. Lin et al. argued that medical students, as novices in medical practice, experience greater physical demands resulting from their lack of efficiency or familiarity with the workload, leading to frustration in learning and reducing their compassion satisfaction [37]. Therefore, understanding of clinical learning process and essential supervision by clinicians in various disciplines might lead to a stress-free learning environment for our students in patient care and hospital settings.

In the present study, we identified a combination of factors associated with depression. In univariate analysis, students who perceived their family income to be insufficient were about three times more likely to develop depressive symptoms than other students. This association has been reported by many authors [36,38]. However, our result failed to determine a significant correlation between depression and family income at the multivariate level when adjusting other confounding factors. A previous study in Saudi Arabia suggested an insignificant association between financial income and the likelihood of depression due to the subjectivity of income estimation [38].

The present study showed that family members’ loss and having psychological illnesses were associated with depression. However, at the multivariate level, the loss of a family member remained a significant depression indicator. Likewise, studies in Saudi Arabia linked depressive symptoms with physiological illness [2,9] and the loss of a family member [2]. Furthermore, inconsistent with a previous study in Saudi Arabia [39], we did not find significant associations between depression and parents’ educational levels.

In the present study, medical students who had difficulties in personal relationships were about two and a half times more likely to develop depression. This might be attributed to the heavy academic requirements of studying medicine, which leave no time for building good personal relationships with friends or society. The other possible reason is that many students at UBCOM who leave their home base could fail to adapt to their new society and environments. Previous studies related the increasing rate of mental disorders to the feeling of isolation from family and community [20,40]. The high rate of depression observed among students with difficulties in personal relationships highlighted the importance of social support from peers, faculty members, and students’ academic counseling. However, students’ focus group discussions might be an essential approach to figure out their essential needs.

Several studies have evaluated the contribution of depression-related factors to the academic status of the medical institution [2,38]. In our findings, students who perceived a lack of college facilities were about three times more depressed than other students. Likewise, a study conducted at King Saud University in Riyadh found that medical students with negative perceptions about the educational environment had high depressive symptoms [38]. Others have suggested that well-structured learning and living environments play an essential role in good mental health [41]. Therefore, promoting the quality and quantity of medical school infrastructure of learning facilities, classroom spaces, laboratories and training sites can reduce students’ depressive symptoms.

Evidence indicated that medical school students could experience significant psychological stress due to substantial academic requirements [2,42]. In the present study, the regret of studying medicine is a persistent and strong factor causing depression. However, having a regretful feeling about studying medicine was a correlate of depression in many studies [4,39]. This can be explained by the fact that many students might find curriculum difficulties and find the nature of studying medical school more demanding than expected. However, our study found a significant correlation between depression and heavy academic load. Another possible factor could be the lack of interest and motivation of students after joining medical school. Research showed that many students select a medical career because of family pressure rather than their own interest [43]. Therefore, understanding the factors influencing students to choose medicine as a career needs to be investigated. Furthermore, implementing proper quality assurance procedures along with students’ perceptions is recommended to evaluate medical curriculum content, teaching and learning strategies.

Regarding academic performance, the proportion of depression was significantly increased among students who perceived that their academic grade was lower than expected. Similar findings have been observed in Korea [34] and India [44]. Yoon et al. found that academic achievement was significantly related to the mean PHQ-9 score, and the prevalence of depression was significantly higher in poorly-perceived academic achievers than in excellent or fair achievers [34]. On the contrary, another study assumed that higher academic achievers might be under massive stress due to the competitive nature of medical school [44]. The present study also found a strong correlation between academic failure and depression, confirming previous findings [45]. Research evidence indicated that depressive symptoms among medical and non-medical students were linked to frequent course failure and lower average curricular grade [46]. Noticeably, our medical students who failed in at least one academic year or a particular course were about three times more depressed than those who did not fail during their studies. Such a situation calls for adopting a mentorship program combined with academic counseling and psychiatric services to guide students toward academic excellence.

The study reveals several limitations that need to be considered. Firstly, this was a cross-sectional design that used a self-assessment measure without any confirmation from clinical physicians. Secondly, depressive symptoms were identified based on a PHQ-9≥10 score; therefore, the correlation between risk factors and the severity of depression was not necessarily identified clearly. Thirdly, the study did not include students who dropped out or who were absent during the survey, which might affect the depression rate among our medical students. Finally, although the evidence in Saudi Arabia suggests that depressive symptoms are higher among female medical students than their male colleagues [2,38], the present study assessed only male students; in fact, the medical program for the females at UBCOM had not yet started.

Despite these limitations, our study offers valuable insights into the relationship between sociodemographic factors and depressive symptoms among male medical students. Recognizing these constraints, we advocate for future research endeavors that address these limitations and contribute to a more nuanced understanding of mental health within the medical education landscape. This is a period that warrants particular attention, as the presence of COVID-19 has the potential to impact the onset of depressive episodes.

## 5. Conclusions

The study concluded that male medical students at UBCOM experience the existence of depressive symptoms. Various correlates of depression related to students’ social lives and academic functioning were identified in this study. Such situations may require more careful attention from medical schools and university administration to prevent and detect depression. However, the institution should take action regarding the criteria for medical student selections and intake, regularly update the medical curriculum, allocate resources, including infrastructure and faculty development, and provide suitable support for junior and senior medical students. Overall, this study serves as a foundation for further exploration in the realm of mental health research, emphasizing the need for holistic investigations into the factors influencing depressive episodes.

## Figures and Tables

**Table 1:** Univariate regression analyzing the associations between sociodemographic and academic factors and depression.

	Variable	n (%)	No. of Students with Depression	COR	*p* Value
1	Family residency			1.165 (0.597–2.271)	0.654
	Bisha province	118 (62.1)	33 (28.0)		
	Another province	72 (37.9)	18 (25.0)		
2	Nature of residency during the study			2.096 (0.905–4.858)	0.080
	With family or friends	143 (75.3)	43 (30.1)		
	Alone	47 (24.7)	08 (17.0)		
3	Father education status			0.773 (0.404–1.479)	0.437
	University degree and above	113 (59.5)	28 (24.8)		
	Below University degree	77 (40.5)	23 (29.9)		
4	Mother education status			1.127 (0.593–2.142)	0.716
	University degree and above	89 (46.5)	25 (28.1)		
	Below University degree	101 (53.2)	26 (25.7)		
5	Satisfaction with family income			2.747 (1.173–6.432)	0.017
	Satisfied	164 (86.3)	39 (23.8)		
	Unsatisfied	26 (13.7)	12 (46.2)		
6	Parental relationship			0.441 (0.174–1.120)	0.079
	Stable	169 (88.9)	42 (24.9)		
	Unstable	21 (11.1)	9 (42.9)		
7	Loss of family members during last month			2.91 (1.193–7.101)	0.015
	Yes	23 (12.1)	11 (47.8)		
	No	167 (87.9)	40 (24.0)		
8	Having, or having a family member with psychological illness			2.581 (1.039–6.413)	0.036
	Yes	22 (11.6)	10(45.5)		
	No	168 (88.4)	41(24.4)		
9	Limited time for social activities			1.088 (0.501–2.361)	0.832
	Yes	147 (77.4)	40 (27.2)		
	No	43 (22.6)	11 (25.6)		
10	Difficulties in personal relationships			2.942 (1.447–5.981)	0.002
	Yes	45 (23.7)	20 (44.4)		
	No	145 (76.3)	31 (21.4)		
11	Use of stimulant			1.897 (0.931–3.864)	0.075
	Yes	46 (24.2)	17 (37.0)		
	No	144 (75.8)	34 (23.6)		
12	Regret studying medicine			5.245(2.583–10.650)	<0.001
	Yes	49 (25.8)	26 (53.1)		
	No	141 (74.2)	25 (17.7)		
13	Failed an academic year			2.537 (1.293–4.975)	0.006
	Yes	57 (30.0)	23 (40.4)		
	No	133 (70.0)	28 (21.1)		
14	Lower grade than expected			3.020 (1.316–6.928)	0.007
	Yes	132 (69.0)	43 (32.6)		
	No	58 (30.0)	08 (13.8)		
15	Conflict with teacher			2.544 (1.098–5.892)	0.026
	Yes	27 (14.0)	12 (44.4)		
	No	163 (85.7)	39 (23.9)		
16	Deficiency of college facilities			2.751 (1.008–7.508)	0.041
	Yes	153 (80.5)	46 (30.1)		
	No	37 (19.4)	05 (13.5)		
17	Heavy academic load			2.310 (1.092–4.888)	0.026
	Yes	125 (65.7)	40 (32.0)		
	No	65 (34.2)	11 (16.9)		

**Table 2:** Multivariate regression analyzing the correlates of depressive symptoms among medical students at the College of Medicine, University of Bisha.

Variable	AOR	*p* Value
Satisfaction with family income		
Satisfied	2.393 (0.864–6.628)	0.093
Unsatisfied	Reference	
Loss of family members during last month		
Yes	**3.69 (1.86–7.413)**	**0.001**
No	Reference	
Having, or having a family member with psychological illness		
Yes	2.817 (0.927–8.559)	0.068
No	Reference	
Difficulties in personal relationships		
Yes	**2.371 (1.009–5.575)**	**0.048**
No	Reference	
Regretting studying medicine		
Yes	**3.764 (1.657–8.550)**	**0.002**
No	Reference	
Failing an academic year		
Yes	**2.559 (1.112–5.887)**	**0.027**
No	Reference	
Lower grade than expected		
Yes	2.556 (0.965–6.767)	0.059
No	Reference	
Conflict with teacher		
Yes	1.622 (0.581–4.524)	0.355
No	Reference	
Deficiency of college facilities		
Yes	1.664 (0.518–5.353)	0.393
No	Reference	
Heavy academic load		
Yes	1.206 (0.492–2.954)	0.683
No	Reference	

## Data Availability

The datasets analysed during the current study are available from the corresponding author on reasonable request.

## Abbreviations

AOR: Adjusted Odd Ratio; CI: Confidence Interval; COR: Crude Odd Ratio; PHQ-9: Patient Health Questionaire-9; SPSS: Statistical Package for Social Sciences; UBCOM: University of Bisha, College of Medicine.

## References

[1] Ngasa S.N., Sama C., Dzekem B.S., Nforchu K.N., Tindong M., Aroke D., Dimala C.A. (2017). Prevalence and Factors Associated with Depression among Medical Students in Cameroon: A Cross-Sectional Study. BMC Psychiatry.

[2] Hamasha A.A.H., Kareem Y.M., Alghamdi M.S., Algarni M.S., Alahedib K.S., Alharbi F.A. (2019). Risk Indicators of Depression among Medical, Dental, Nursing, Pharmacology, and Other Medical Science Students in Saudi Arabia. Int. Rev. Psychiatry.

[3] Agyapong-Opoku G., Agyapong B., Obuobi-Donkor G., Eboreime E. (2023). Depression and Anxiety among Undergraduate Health Science Students: A Scoping Review of the Literature. Behav. Sci..

[4] Njim T., Mbanga C.M., Tindong M., Fonkou S., Makebe H., Toukam L., Fondungallah J., Fondong A., Mulango I., Kika B. (2019). Burnout as a Correlate of Depression among Medical Students in Cameroon: A Cross-Sectional Study. BMJ Open.

[5] Peng P., Hao Y., Liu Y., Chen S., Wang Y., Yang Q., Wang X., Li M., Wang Y., He L. (2023). The Prevalence and Risk Factors of Mental Problems in Medical Students during COVID-19 Pandemic: A Systematic Review and Meta-Analysis. J. Affect. Disord..

[6] Alkot M.M., Alnewirah A.Y., Bagasi A.T., Alshehri A.A., Bawazeer N.A. (2017). Depression among Medical versus Non-Medical Students in Umm Al-Qura University, Makkah Al-Mukaramah, Saudi Arabia. Am. J. Psychiatry Neurosci..

[7] Mayer F.B., Santos I.S., Silveira P.S.P., Helena M., Lopes I., Regina A., Dias N., Campos E.P., Andrade B., De Abreu L. (2016). Factors Associated to Depression and Anxiety in Medical Students: A Multicenter Study. BMC Med. Educ..

[8] Puthran R., Zhang M.W.B., Tam W.W., Ho R.C. (2016). Prevalence of Depression amongst Medical Students: A Meta-Analysis. Med. Educ..

[9] Albajjar M.A., Bakarman M.A. (2019). Prevalence and Correlates of Depression among Male Medical Students and Interns in Albaha University, Saudi Arabia. J. Fam. Med. Prim. Care.

[10] Hakami R.M. (2018). Prevalence of Psychological Distress Among Undergraduate Students at Jazan University: A Cross-Sectional Study. Saudi J. Med. Med. Sci..

[11] El-Gilany A.H., Amr M.H.S. (2008). Perceived Stress among Male Medical Students in Egypt and Saudi Arabia: Effect of Sociodemographic Factors. Ann. Saudi Med..

[12] Bin Abdulrahman K.A., Saleh F. (2015). Steps towards Establishing a New Medical College in the Kingdom of Saudi Arabia: An Insight into Medical Education in the Kingdom. BMC Med. Educ..

[13] Bedaso A., Kediro G., Yeneabat T. (2018). Factors Associated with Depression among Prisoners in Southern Ethiopia: A Cross-sectional Study. BMC Res. Notes.

[14] Nguyen T., Nguyen N., Pham M.V., Van Pham H., Nakamura H. (2018). The Four-Domain Structure Model of a Depression Scale for Medical Students: A Cross-Sectional Study in Haiphong, Vietnam. PLoS ONE.

[15] Dhanoa S., Oluwasina F., Shalaby R., Kim E., Agyapong B., Hrabok M., Eboreime E., Kravtsenyuk M., Yang A., Nwachukwu I. (2022). Prevalence and Correlates of Likely Major Depressive Disorder among Medical Students in Alberta, Canada. Int. J. Environ. Res. Public Health.

[16] Mhata N.T., Ntlantsana V., Tomita A.M., Mwambene K., Saloojee S. (2023). Prevalence of Depression, Anxiety and Burnout in Medical Students at the University of Namibia. S. Afr. J. Psychiatry.

[17] Aleisa M.A., Abdullah N.S., Alqahtani A.A.A., Aleisa J.A.J., Algethami M.R., Alshahrani N.Z. (2022). Association between Alexithymia and Depression among King Khalid University Medical Students: An Analytical Cross-Sectional Study. Healthcare.

[18] Bert F., Lo Moro G., Corradi A., Acampora A., Agodi A., Brunelli L., Chironna M., Cocchio S., Cofini V., D’Errico M.M. (2020). Prevalence of Depressive Symptoms among Italian Medical Students: The Multicentre Cross-Sectional “PRIMES” Study. PLoS ONE.

[19] Fuchs S., Stoevesandt D., Sapalidis A., Rehnisch C., Watzke S. (2022). Prevalence and Predictive Factors for Depressive Symptoms among Medical Students in Germany—A Cross-Sectional Study Methods: A Total Number of N = 1103 Medical Students of a Middle-Sized German University Were Sampled and Surveyed Regarding Depressive. GMS J. Med. Educ..

[20] Kumar G.S., Jain A., Hegde S. (2020). Prevalence of Depression and Its Associated Factors Using Beck Depression Inventory among Students of a Medical College in Karnataka. Indian J. Psychiatry.

[21] Peluso M.J., Guille C., Sen S., Mata D.A. (2017). Prevalence of Depression, Depressive Symptoms, and Suicidal Ideation Among Medical Students: A Systematic Review and Meta-Analysis. JAMA.

[22] Aljadani A., Alsolami A., Almehmadi S., Alhuwaydi A. (2021). Epidemiology of Burnout and Its Association with Academic Performance among Medical Students at Hail University, Saudi Arabia. Sultan Qaboos Univ. Med. J..

[23] Ibrahim M.E. (2020). Team-Based Learning Student Assessment Instrument (TBL-SAI) for Assessing Students’ Acceptance of TBL in a Saudi Medical School. Psychometric Analysis and Differences by Academic Year. Saudi Med. J..

[24] Ibrahim M.E., Al-Shahrani A.M., Abdalla M.E., Abubaker I.M., Mohamed M.E. (2018). The Effectiveness of Problem-Based Learning in Acquisition of Knowledge, Soft Skills During Basic and Preclinical Sciences: Medical Students’ Points of View. Acta Inform. Med..

[25] Ibrahim M., Al-Shahrani A. (2018). Implementing of a Problem-Based Learning Strategy in a Saudi Medical School: Requisites and Challenges. Int. J. Med. Educ..

[26] Salih K.M., Elnour S., Mohammed N., Alkhushayl A.M., Alghamdi A.A., Eljack I.A., Al-Faifi J., Ibrahim M.E. (2023). Climate of Online E-Learning During COVID-19 Pandemic in a Saudi Medical School: Students’ Perspective. J. Med. Educ. Curric. Dev..

[27] Al-Khani A.M., Sarhandi M.I., Zaghloul M.S., Ewid M., Saquib N. (2019). A Cross-Sectional Survey on Sleep Quality, Mental Health, and Academic Performance among Medical Students in Saudi Arabia. BMC Res. Notes.

[28] Al Bahhawi T., Albasheer O.B., Mohammed A., Mohammed O. (2018). Depression, Anxiety, and Stress and Their Association with Khat Use: A Cross-Sectional Study among Jazan University Students, Saudi Arabia. Neuropsychiatr. Dis. Treat..

[29] Qin X., Wang S., Hsieh C.R. (2018). The Prevalence of Depression and Depressive Symptoms among Adults in China: Estimation Based on a National Household Survey. China Econ. Rev..

[30] Alhadi A.N., Alateeq D.A., Al-Sharif E., Bawazeer H.M., Alanazi H., Alshomrani A.T., Shuqdar R.M., Alowaybil R. (2017). An Arabic Translation, Reliability, and Validation of Patient Health Questionnaire in a Saudi Sample. Ann. Gen. Psychiatry.

[31] Inoue T., Tanaka T., Nakagawa S., Nakato Y., Kameyama R., Boku S., Toda H., Kurita T., Koyama T. (2012). Utility and Limitations of PHQ-9 in a Clinic Specializing in Psychiatric Care. BMC Psychiatry.

[32] Beshr M.S., Beshr I.A., Al-Qubati H. (2024). The Prevalence of Depression and Anxiety among Medical Students in Yemen: A Cross-Sectional Study. J. Affect. Disord..

[33] Vankar J.R., Prabhakaran A., Sharma H. (2014). Depression and Stigma in Medical Students at a Private Medical College. Indian J. Psychol. Med..

[34] Yoon S., Lee Y., Han C., Steffens D.C., Kim Y. (2014). Usefulness of the Patient Health Questionnaire-9 for Korean Medical Students. Acad Psychiatry.

[35] Nahas A.R.M.F., Elkalmi R.M., Al-Shami A.M., Elsayed T.M. (2019). Prevalence of Depression among Health Sciences Students: Findings from a Public University in Malaysia. J. Pharm. Bioallied. Sci..

[36] Roh M., Jeon H.J., Kim H. (2010). The Prevalence and Impact of Depression among Medical Students: A Nationwide Cross-Sectional Study in South Korea. Acad. Med..

[37] Lin Y.K., Lin C., Lin B.Y., Chen D. (2019). Medical Students’ Resilience: A Protective Role on Stress and Quality of Life in Clerkship. BMC Med. Educ..

[38] Al-Faris E.A., Irfan F., Van der Vleuten C.P.M., Naeem N., Alsalem A., Alamiri N., Alraiyes T., Alfowzan M., Alabdulsalam A., Ababtain A. (2012). The Prevalence and Correlates of Depressive Symptoms from an Arabian Setting: A Wake up Call. Med. Teach..

[39] Ibrahim N., Kharboush D.A.-, El-khatib L., Al A., Asali D. (2013). Prevalence and Predictors of Anxiety and Depression among Female Medical Students in King Abdulaziz University, Jeddah, Saudi Arabia. Iran. J. Public Health.

[40] Goebert D., Thompson D., Takeshita J., Beach C., Bryson P., Ephgrave K., Kent A., Kunkel M., Schechter J., Tate J. (2009). Depressive Symptoms in Medical Students and Residents: A Multischool Study. Acad. Med..

[41] Deb S., Banu P.R., Thomas S., Vardhan R.V., Rao P.T., Khawaja N. (2016). Depression among Indian University Students and Its Association with Perceived University Academic Environment, Living Arrangements and Personal Issues. Asian J. Psychiatr..

[42] Azim S.R., Baig M. (2017). Frequency and Perceived Causes of Depression, Anxiety and Stress among Medical Students of a Private Medical Institute in Karachi: A Mixed Method Study. J. Pak. Med. Assoc..

[43] Shankar N., Singh S., Gautam S., Dhaliwal U. (2013). Motivation and Preparedness of First Semester Medical Students for a Career in Medicine. Indian J. Physiol. Pharmacol..

[44] Sidana S., Kishore J., Ghosh V., Gulati D., Jiloha R.C., Anand T. (2012). Prevalence of Depression in Students of a Medical College in New Delhi: A Cross-Sectional Study. Australas. Med. J..

[45] Waqas A., Rehman A., Malik A., Muhammad U., Khan S., Mahmood N. (2015). Association of Ego Defense Mechanisms with Academic Performance, Anxiety and Depression in Medical Students: A Mixed Methods Study. Cureus.

[46] Sousa J.M.D.E., Moreira C.A., Telles-Correia D. (2018). Anxiety, Depression and Academic Performance: A Study Amongst Portuguese Medical Students Versus Non- Medical Students. Acta Med. Port..

